# A Review of Evaluations of Electronic Event-Based Biosurveillance Systems

**DOI:** 10.1371/journal.pone.0111222

**Published:** 2014-10-20

**Authors:** Kimberly N. Gajewski, Amy E. Peterson, Rohit A. Chitale, Julie A. Pavlin, Kevin L. Russell, Jean-Paul Chretien

**Affiliations:** 1 Response Directorate, Federal Emergency Management Agency, Washington, DC, United States of America; 2 Division of Integrated Biosurveillance, Armed Forces Health Surveillance Center, Silver Spring, MD, United States of America; 3 Headquarters, Armed Forces Health Surveillance Center, Silver Spring, MD, United States of America; Global Disease Detection-Kenya, United States of America

## Abstract

Electronic event-based biosurveillance systems (EEBS’s) that use near real-time information from the internet are an increasingly important source of epidemiologic intelligence. However, there has not been a systematic assessment of EEBS evaluations, which could identify key uncertainties about current systems and guide EEBS development to most effectively exploit web-based information for biosurveillance. To conduct this assessment, we searched PubMed and Google Scholar to identify peer-reviewed evaluations of EEBS’s. We included EEBS’s that use publicly available internet information sources, cover events that are relevant to human health, and have global scope. To assess the publications using a common framework, we constructed a list of 17 EEBS attributes from published guidelines for evaluating health surveillance systems. We identified 11 EEBS’s and 20 evaluations of these EEBS’s. The number of published evaluations per EEBS ranged from 1 (Gen-Db, GODsN, MiTAP) to 8 (GPHIN, HealthMap). The median number of evaluation variables assessed per EEBS was 8 (range, 3–15). Ten published evaluations contained quantitative assessments of at least one key variable. No evaluations examined usefulness by identifying specific public health decisions, actions, or outcomes resulting from EEBS outputs. Future EEBS assessments should identify and discuss critical indicators of public health utility, especially the impact of EEBS’s on public health response.

## Introduction

Approximately 65% of the world’s first news about infectious disease events comes from informal sources, such as the internet, and almost all major outbreaks investigated by the World Health Organization (WHO) are first identified through these informal sources [1]–[3]. Electronic event-based biosurveillance uses information on events impacting human health or the economy from internet sources, simultaneously incorporating diverse streams of data [4]. Electronic event-based biosurveillance systems (EEBS’s) are an increasingly important source of epidemiologic intelligence [1]–[2].

Rapidly expanding worldwide access to the internet has fueled an increase in the number, and popularity, of EEBS’s. There are several benefits to these new forms of surveillance. Many EEBS’s allow citizens to report public health events via social media platforms or electronic communication channels independently of governments. Governments are no longer in sole control of their public health information, making it substantially harder to hide or delay outbreak or event reports [3]. Additionally, since protocols and confirmatory testing requirements do not delay the reports, they are considerably timelier than traditional surveillance sources [3]. Another beneficial aspect of EEBS’s is that many are publicly accessible. All subscribers have equally timely access to breaking reports regardless of their public health affiliations.

However, the same aspects of EEBS’s that make them important new surveillance tools also may make them less reliable tools. Because many sources of data are not verified by public health professionals, these systems are prone to noise and false alarms [2]. Several researchers have commented that EEBS’s especially tend to lack specificity in their alerts and reports [4]–[6]. Many EEBS’s also face challenges in interoperability, scalability, population coverage, and interface customizability [4].

There have been evaluations of individual EEBS’s, but there have not been structured evaluations of multiple EEBS’s, or a comprehensive assessment of all EEBS evaluations. Our objective was to assess evaluations of EEBS’s, and to recommend criteria for future evaluations. Our findings may help guide future EEBS development to most effectively exploit web-based information for biosurveillance.

## Methods

We consulted an EEBS inventory [7] and biosurveillance experts (via informal queries to staff within our organization) to identify EEBS’s that use publicly available internet information sources, include events that impact human health, and have global scope. We excluded systems that did not include infectious disease events.

To construct an evaluation framework for EEBS’s, we reviewed the Centers for Disease Control and Prevention (CDC) surveillance system evaluation guidelines [8] and CDC evaluation guidelines for outbreak detection systems [9]. From those guidelines we selected evaluation variables highlighted as of primary importance in one of the guidelines, or mentioned as of secondary importance in both guidelines. We combined variables that are highly similar, narrowing the list to 17 variables: acceptability, accessibility, cost, data quality, flexibility, population coverage, predictive value positive, purpose, portability, representativeness, resources needed, sensitivity, simplicity, stability, timeliness, usefulness, and validity.

We searched PubMed and Google Scholar for publications with the name of one or more of the included EEBS’s in any search field. We included structured evaluations of the systems as well as system descriptions if they discussed the system’s performance with respect to one of the evaluation variables, even if that discussion was not a structured evaluation. For each evaluation, we recorded the evaluation variables discussed for each EEBS, and determined whether the evaluation assessed the variable quantitatively or qualitatively.

## Results

We identified 11 EEBS’s meeting the inclusion criteria (Table 1) [1]–[2],[4]–[6],[10]–[22]. The oldest system was ProMed, founded in 1994 and the newest system was Geni-Db, founded in 2012. The systems used automation to varying degrees in extracting information from the internet, processing it, and producing reports or alerts. For example, some EEBS’s relied heavily on subject matter experts to assess reports from various sources (e.g., ProMED) or on manual translation by linguists with regional expertise (e.g., Argus); others used automated procedures for posting and mapping (e.g., HealthMap) or translation (e.g., GPHIN).

**Table 1:** Number of published evaluations and variables on identified EEBS’s.

EEBS*	Yearstarted	Description	No. evaluations	No. key variablesassessed
Argus	2005	Manual translation of news reports bylinguists with regional expertise	5 [2],[4],[10]–[12]	7
BioCaster(http://born.nii.ac.jp)	2006	Automated text mining of RSS newsfeeds	5 [2],[12]–[15]	9
EpiSpider	2006	Automated conversion of topic and locationdata for online event reports (e.g., ProMED)to RSS feeds	2 [2],[15]	4
Geni-Db(http://born.nii.ac.jp/_dev/static/genidb)	2012	Extracts event data from Biocaster and providesin searchable tables	1 [16]	4
GODSn	2006	Natural language processing and mappingfor RSS news feeds	1 [11]	3
GPHIN(http://www.who.int/csr/alertresponse/epidemicintelligence/en)	1997	Automated translation and classification ofreports from news feed aggregators withanalyst decision to alert	7 [1]–[2],[4],[6],[10],[17],[18]	10
HealthMap(http://www.healthmap.org/en)	2006	Automated processing and mapping ofreports from RSS feeds and other onlinesources (e.g., official reports)	7 [2],[4]–[5],[15],[18]–[20]	12
MedISys(http://medusa.jrc.it/medisys/homeedition/en/home.html)	2006	Automated processing of news source reportswith email alerting	2 [4],[21]	4
MiTAP	2001	Automated translation and processing of onlinereports	1 [22]	5
ProMed(http://www.promedmail.org)	1994	Manual screening/posting of reports fromvarious sources (e.g., media, official reports,local observations)	5 [4]–[6],[17]–[18]	12
PULS(http://puls.cs.helsinki.fi/static/index.html)	2006	Extracts event data from MedISys and providesin searchable tables	2 [4],[21]	5

*Not all EEBS’s were operational at the time of this report. URL provided when one could be identified.

Older systems had more evaluations than the newer systems, with the exception of HealthMap, which ranked second for the most evaluations despite being founded in 2006. The median number of key variables assessed per EEBS was 8 (range, 3–15), with 6 evaluations assessing 7 or more key variables. Older systems were more likely to be reviewed in parallel with each other. There were two or fewer published evaluations on the GODsN, EpiSpider, MiTAP and Geni-Db systems.

**Figure 1:**
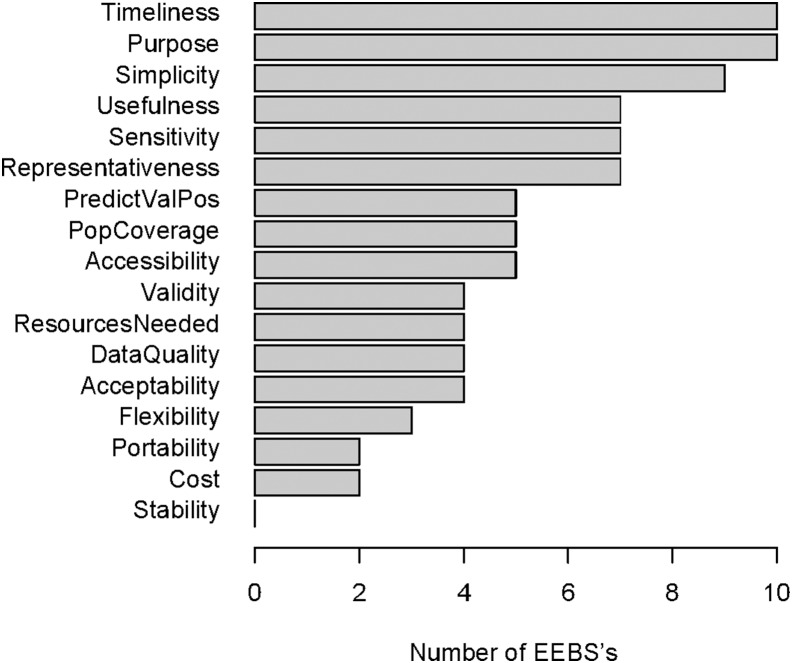
Assessment of variables across EEBS evaluations.

Ten of 20 published evaluations contained quantitative assessments of at least one key variable, while the others mentioned evaluation variables but did not provide results reflective of a systematic assessment of those variables. Timeliness and purpose were assessed for 10 of 11 EEBS’s, while data quality and validity were assessed for 4 of 11 EEBS’s (Figure 1).

Nine evaluations assessed usefulness for 7 EEBS’s by citing instances where the EEBS detected an outbreak earlier than other surveillance systems, or by eliciting user feedback, but none identified specific public health decisions, actions, or outcomes resulting from EEBS outputs. No evaluations examined system stability, and only two systems were evaluated on cost.

Because of the lack of detail provided for evaluations on key variables, it was not possible to determine which EEBS’s have been most useful or which EEBS approaches are most promising.

## Discussion

We found a paucity of evaluation results for EEBS’s on key evaluation variables, with only half of published evaluations reporting quantitative assessments of at least one key variable. Many evaluations mentioned key variables only in passing, and did not present results suggesting that a systematic quantitative or qualitative assessment was performed.

Timeliness, a possible advantage of EEBS’s compared to traditional surveillance systems, was assessed for 10 of 11 EEBS’s, but data quality and validity, for which EEBS’s may face more challenges, were infrequently assessed (4 out of the 11 EEBS’s). Perhaps most importantly, no evaluations cited specific examples of public health decisions, actions, or outcomes resulting from EEBS alerts. To our knowledge, this is the first comprehensive assessment of the evaluation literature for EEBS’s, and provides a snapshot of the current knowledge of EEBS performance characteristics and overall usefulness.

We note two important limitations of this study. First, we focused on global-scale EEBS’s. While these may be of broad interest to the public health community, we cannot comment on the extent of local or regional-scale EEBS evaluation. Evaluations of these systems may provide useful lessons for global-scale EEBS’s. Second, we limited the assessment to peer-reviewed, published evaluations. This approach likely does not capture all EEBS evaluations, though some excluded evaluations may be difficult to access or of less-certain quality.

Future EEBS evaluations should identify and discuss critical indicators of public health utility, using quantitative or qualitative approaches to assess the usefulness of EEBS’s in guiding public health action. They should also assess the novel aspects of EEBS’s compared to traditional surveillance approaches, and include variables of special interest to potential EEBS users such as policy readiness, number and geographic profiles of users, number of sources, system redundancy, and input/output geography [23]; explore benefits of participatory biosurveillance and analytical tools, which some systems offer [24]; and consider ways of integrating outputs of various EEBS’s to combine their respective strengths. Initial investigations into the effects of combining systems by Barboza et al. [25] have concluded that significant value can be added and synergistic effects can be observed. Further investigations into the value added by combining systems need to be explored, particularly any improvements in sensitivity, predictive value positive and usefulness.

We urge developers and users to conduct and publish evaluations of EEBS’s. While they clearly offer powerful biosurveillance capabilities complementing traditional surveillance approaches, further indications of how and under what circumstances they are most useful, based on real-world experience, could advance EEBS development and effective integration into public health programs.
